# Prediction of sepsis in trauma patients

**DOI:** 10.4103/2321-3868.135479

**Published:** 2014-07-28

**Authors:** He Jin, Zheng Liu, Ya Xiao, Xia Fan, Jun Yan, Huaping Liang

**Affiliations:** State Key Laboratory of Trauma, Burns and Combined Injury, Research Institute of Surgery, Daping Hospital, The Third Military Medical University, Chongqing, 400042 China

**Keywords:** Sepsis, trauma, infection, prediction

## Abstract

Trauma is one of the leading causes of death worldwide. Approximately 39.5% of deaths occur in the hospital, and the mortality rate of delayed death caused by septic complications is still high. Early prediction of the development of sepsis can help promote early intervention and treatment for patients and contribute to improving patient outcomes. Thus so far, biomarkers, patient demographics and injury characteristics are the main methods used for predicting sepsis in trauma patients. However, studies that verify their predictive value are limited, and the results are still controversial. More work should be conducted to explore more efficient and accurate ways to predict post-traumatic sepsis.

## Introduction

Trauma is still a leading cause of death worldwide, with a mortality of 5,800,000 people per year.[1] Fifty-three percent of deaths that happen after trauma occur at the scene of the accident, 7.5% occur in the emergency department, and 39.5% occur in the hospital.[2] Patients who survive the early period after trauma may suffer morbidity and complications in succeeding treatment phases.[3] The initial injury and subsequent operative treatments promote a pro-inflammatory response, which is exaggerated and may cause organ injury (acute respiratory distress syndrome [ARDS] and multiple organ failure [MOF]).[4] Meanwhile, an anti-inflammatory response is involved in reducing the potentially harmful effects of the pro-inflammatory response and enhances susceptibility to secondary infections, which increases the risk of sepsis and septic complications (ARDS, MOF).[4,5] Although the incidence of post-traumatic sepsis in the hospital has decreased in the past two decades,[6] the mortality (between 19.5% and 23%) of septic trauma patients is still high.[6,7] Early diagnosis and treatment of these patients with antibiotics can improve the prognosis and reduce mortality.[8–11]

**Table:** 

Access this article online	
**Quick Response Code**:	**Website**: www.burnstrauma.com
	**DOI**: 10.4103/2321-3868.135479

Diagnosis of the pathogen that causes sepsis requires bacterial culture,[12] but it is often delayed due to long culture times (24 h to 48 h). Furthermore, a third or more of septic patients with infections have cultures that are negative for bacteria.[11,13–17] This may contribute to the high mortality. Identification of the factors associated with the development of post-traumatic sepsis may help in the early prediction of the occurrence of this complication so that timely interventions could be performed to improve the outcomes. Research on some factors, such as biomarkers, patient demographics and injury characteristics have revealed some elements that are predictable in post-traumatic sepsis. Here, we review these predictors and risk factors of post-traumatic sepsis.

## Biomarkers

According to the Biomarkers Definitions Working Group, a biomarker is a characteristic that is objectively measured and evaluated as an indicator of normal biological processes, pathogenic processes or pharmacologic responses to a therapeutic intervention.[18] In other words, biomarkers are tools to measure biologic homeostasis that give standard to what is normal and provide a quantifiable method for predicting or detecting what is abnormal.[19] The ideal biomarker for sepsis should have a high sensitivity allowing for early diagnosis and would be specific for pathogenic microorganisms to allow appropriate therapy.[20] It has been reported that more than 80 molecules have been proposed as useful biomarkers of sepsis,[21] and to date, the number has increased to 178 or more.[22] To be considered a valid biomarker, 3 aspects must be present: (1) proving that the test truly measures a particular molecular species or its relevant biological activity; (2) proving that measurement of the biomarker discriminates patients with a disease from those who are without the disease; (3) proving that measurement of the biomarker can inform a clinical decision that can improve patient outcomes.[23] Here, we list some representable candidates among the potential biomarkers of post-traumatic sepsis.

### Procalcitonin (PCT)

PCT is a precursor of the hormone calcitonin, which is codified by the CALC-I gene located on chromosome 11 and is produced and secreted by parafollicular C cells of the thyroid to sustain calcium homeostasis.[24] PCT has been shown to be a marker of bacterial infection and sepsis[25,26] as PCT is released systemically from various types of cells outside the thyroid as a response to bacterial infection.[27] On the condition of systemic bacterial infection or by stimulation with endotoxin or proinflammatory cytokines such as tumor necrosis factor (TNF)-α, interleukin (IL)-6 and IL-1, PCT levels increase 1,000 times within a few hours.[28,29] The half-life of PCT, approximately 22 h, is another characteristic that can be used as a biomarker for bacterial infection. Its levels show a rapid decrease when infection is resolved, whereas many other inflammatory biomarkers still have high levels during the acute-phase response.[28] For predicting post-traumatic sepsis, studies have shown the rapid kinetics of PCT, with levels peaking at 24–48 h after trauma and rapid decrease in non-complicated patients, whereas with constant high levels in septic patients.[4] Continuous high levels or secondary increases of PCT are predictors of sepsis.[27,28,30–34] PCT as a biomarker is useful in the prediction and early diagnosis of sepsis in trauma patients. Currently, PCT is already used in clinical practice, and it is used to guide antibiotic therapy in patients with lower respiratory tract infections or other infections such as fungal infections, postoperative fever and suspected bloodstream infections.[35]

### C-reactive protein (CRP)

CRP belongs to the acute phase protein family. Each one is made of 5 protomers of 206 amino acid residues, and belongs to the pentraxin family of calcium-dependent ligand-binding plasma proteins.[36] CRP is mainly synthesized in the hepatocytes, and its transcription is reduced by the cytokine IL-6, which is predominantly released by macrophages in response to various types of systemic inflammation, including infections or trauma.[36–38] Therefore, it is a sensitive marker of inflammation and tissue damage. The half-life of CRP is 19 h.[39] Serum CRP is used as a biomarker because of the rapid concentrations that increase in response to inflammation, the shorter half-life and the widely available inexpensive test. Many researchers have explored the predictive value of CRP for post-traumatic sepsis, but the results are unsatisfactory. Both prospective studies and retrospective studies have reported no predictive power of CRP for sepsis in trauma patients.[4,28,30–32,40–43]

### IL-6

IL-6 is a glycoprotein synthesized by various types of cells including T- and B-cells and endothelial cells. Other cytokines (IL-1, TNF-α), viruses and bacterial components, such as lipopolysaccharide (LPS), induce the production of IL-6. IL-6 induces hepatic production of acute-phase proteins such as CRP and complement factors, regulation of B- and T-lymphocytes, differentiation of cytotoxic T-cells, and an enhanced activity of natural killer (NK) cells.[44] Its release is triggered by tissue damage or infection. It is a cytokine involved in both pro-inflammatory and anti-inflammatory responses.[45] IL-6 has a rapid onset, peaking within 2 h after the infectious stimulus.[46] The results of studies on the predictive value of IL-6 for post-traumatic sepsis are controversial. Some studies have found that IL-6 is able to discriminate trauma patients prone to sepsis[31,32] while others have shown no correlation between the IL-6 levels and sepsis development.[42,43,47–50]

### IL-10

IL-10 is a protein produced by T-lymphocytes, B-lymphocytes, macrophages and dendritic cells (DC).[51] It is an anti-inflammatory cytokine playing a role in counter inflammatory and autoimmune pathologies.[52] IL-10 downregulates MHC class II and co-stimulatory molecules B7-1/B7-2 expression on monocytes and macrophages, inhibiting their antigen-presenting function, and limits the synthesis of pro-inflammatory cytokines (IL-1, TNF-α) as well as decreases cytokine production of Th-1 cells.[51] IL-10 peaks quickly, within a few hours (4 h) following trauma, and the levels decrease rapidly in all patients (the first day after trauma).[53,54] IL-10 levels have been shown to be significantly higher in patients who develop sepsis at the point of admission.[4,53–56]

### Neopterin

Neopterin is a pteridine produced by monocytes or macrophages upon stimulation with interferon (IFN)-γ and is then released into body circulation.[57] Neopterin is useful for the diagnosis of bacterial and viral infections and systemic inflammation. In addition, increased levels of neopterin are associated with endothelial damage, organ dysfunction and sepsis.[58] Among the studies performed on predicting post-traumatic sepsis, neopterin levels have shown no significant difference between patients who developed and did not develop sepsis.[42,58–60]

### Pancreatic stone protein/regenerating protein (PSP/reg)

PSP/reg is a lectin-binding acute phase protein and was initially found in patients with pancreatitis.[61] PSP/reg acts as an acute phase protein causing the activation of leukocytes and can also be observed in other cells outside the pancreas.[32] Its release is reduced by IL-6 following tissue injury.[62] PSP/reg levels can predict and distinguish septic complications in post-traumatic patients.[32]

### IL-1

IL-1 is an important mediator of innate immunity and inflammation. It can significantly lengthen the lifespan and activate the function of neutrophils and macrophages in response to infections.[63] Its effects on the central nervous system cause fever, the elevated temperature leads to an increased migration of leukocytes. Few studies demonstrate evidence of the predictive power of IL-1 for sepsis after trauma, except Menges *et al.*[55] who reported the positive correlation between IL-1 and sepsis.

### Amino-terminal pro-peptide (NT-proCNP)

NT-proCNP is a part of the natriuretic peptide family and was first identified in 1990. CNP participates in physiological processes such as bone growth, reproduction, nerve growth, and re-endothelialization.[64] ProCNP protein is a precursor of CNP. As a cleavage product of proCNP, Amino-terminal pro-C-type natriuretic peptide (NT-proCNP) is the N-terminal fragment of the C-type natriuretic peptide precursor.[65] The amounts of NT-proCNP are equal to CNP in human plasma and NT-proCNP is considered to be a more reliable indicator of the extent of CNP synthesis.[65] Results of a study show that the levels of circulating NT-proCNP can discriminate poly trauma patients without traumatic brain injury who develop sepsis from those who do not.[66]

### Polymorphonuclear elastase (PMNE)

In healthy adults, polymorphonuclear (PMN) circulates during the resting state and can be activated following major trauma.[67] PMN is the main effector cell of the inflammatory response post-trauma and it produces and releases toxic reactive oxygen species. PMN activation and inflammatory response post-trauma may be reflected in serum elastase levels.[67] Some studies have shown the difference in PMN elastase between patients with and without infection or sepsis[47,59], while others have shown that it has no correlation with post-traumatic infective complications.[42,43]

### Lactate clearance

Persistent occult hypoperfusion is a risk factor for infections following trauma,[68] and lactate clearance is proposed as a measure of early sepsis resuscitation effectiveness.[69] Thus, lactate clearance can be a biomarker of sepsis. During the first 12–24 h, the lactate clearance is associated with post-traumatic sepsis.[31,68]

### IL-18

As a member of the IL-1 cytokine family, IL-18, which is produced by a variety of cells including Kuppfer cells, monocytes, dendritic cells (DC), macrophages, and so on, induces the production of IFN-γ and other cytokines. It is found to have high levels in sepsis patients compared to healthy people.[70] Mommsen *et al.*[58] has proposed IL-18 concentrations as early markers for post-traumatic complications such as sepsis and MODS.

### Monocyte Human Leukocyte Antigen DR (mHLA-DR)

Human leukocyte antigen-DR (HLA-DR) is a member of the MHC class II system. HLA-DR is expressed in antigen presenting cells (APC) including monocytes, macrophages, dendritic cells and B lymphocytes.[71] Low expression of HLA-DR on circulating monocytes (mHLA-DR) is reported as an indicator of post-trauma immune suppression.[71] Studies show that the decreased level of mHLA-DR is a biomarker of sepsis development after major trauma.[72,73]

### Other biomarkers for sepsis following trauma

Some potential biomarkers, such as Toll-like receptor (TLR)-9,[74] PMN cluster of differentiation (CD) 11b,[49] Soluble factor associated suicide (FAS) (sFAS),[47] Group-specific component globulin (Gc-globulin),[75] kynurenine values and kynurenine-tryptophan ratios,[76] and the soluble thrombomodulin (s-TM) level,[77] have also been reported to have predictive abilities for post-traumatic sepsis. TNF-α, an important cytokine, has been shown to have no sufficiently predictive value for sepsis development after trauma.[50]

Evaluation of biomarkers of post-traumatic sepsis is illustrated in Table 1.

**Table 1:** **Evaluation of biomarkers of post-traumatic sepsis**

Biomarkers of post-traumatic sepsis	Predictive value	Study
PCT	Yes	[27,28,30–34]
CRP	No	[4,28,30–32,40–43]
IL-6	Controversial	Yes [31,32]
		No [42,43,47–50]
IL-10	Yes	[4, 53–56]
Neopterin	No	[42, 58–60]
PSP/reg	Yes	[32]
IL-1	Yes	[55]
NT-proCNP	Yes	[66]
PMNE	Controversial	Yes [47, 59]
		No [42, 43]
Lactate clearance	Yes	[31, 68]
IL-18	Yes	[58]
mHLA-DR	Yes	[72, 73]
TNF-α	No	[60]
TLR-9	Yes	[74]
PMN CD11b	Yes	[49]
sFAS	Yes	[47]
Gc-globulin	Yes	[75]
s-TM	Yes	[77]
kynurenine values and kynureninetryptophan ratio	Yes	[76]

PCT = Procalcitonin, CRP = C-reactive protein, IL = Interleukin, PSP/reg = Pancreatic stone protein/regenerating protein, NT-proCNP = Amino-terminal pro-peptide, PMNE = Polymorphonuclear elastase, mHLA-DR = Monocyte Human Leukocyte Antigen DR, TNF-α = Tumor necrosis factor α, TLR-9 = Toll-like receptor-9, PMN CD11b = Polymorphonuclear cluster of differentiation 11b, sFAS = Soluble FAS, Gc-globulin = Group-specific component globulin, s-TM = Soluble thrombomodulin

Until now, many biomarkers have been proposed in the field of sepsis. However, there are only a few biomarkers that have been shown to be useful for predicting post-traumatic sepsis. Among the biomarkers for sepsis following trauma, PCT is the most extensively investigated biomarker, and the results show good application in predicting this complication. However, others, such as CRP, though many studies have been performed on it, show no predictive power for trauma patients. For some biomarkers such as IL-6 and PMNE, the results are controversial. There are also some biomarkers such as IL-1 and IL-18, that show the predictive value of sepsis post-trauma, but the studies are few and the results need further evidence to support this. Traumatic injuries cause great changes in the immunological and neurohormonal environments, which then affect physiological processes. After trauma, the innate immune system is activated, and many pro-inflammatory cytokines, such as TNF-α, IL-1 and IL-6 are released, leading to systemic inflammation. Activation of neutrophils and endothelial cells can cause endothelium and tissue damage. To counter these disadvantages, anti-inflammatory cytokines, such as IL-10, are released, leading to immune suppression and increased risk of secondary infections. The primary cytokines TNF-α and IL-1 can induce the release of following cytokines including IL-6 and IL-8. IL-6 again promotes the production of acute phase proteins such as PCT and CRP.

Most of the biomarkers illustrated above participate in the reaction of systemic inflammation. However, post-trauma physiological processes may become more complicated due to immune function disorders caused by multiple trauma. For example, in the condition of abdominal or brain trauma, the kinetics of biomarkers can be changed.[4] Therefore, more tests should be performed to verify the predictive value of the present biomarkers and to find more biomarkers suitable to predict traumatic sepsis.

### Patient demographics

Patient demographics including age, gender and race are risk factors associated with post-traumatic sepsis [Table 2]. Older age is an independent risk factor for sepsis following trauma.[3,6,78] This may be because elderly trauma patients have decreased cardiopulmonary function, poor nutritional status, and are susceptible to increased bleeding after injuries. These factors may contribute to the disorder of physiological processes and immunologic function. In addition, elder trauma patients may have more pre-existing diseases than young patients, and the pre-existing diseases are also a risk factor for post-traumatic sepsis.[7]

**Table 2:** **Risk factors of patient demographics associted with post-traumatic sepsis**

Patient demographics	Predictive value	Study
Age	Yes	[3, 6, 78]
Gender	Yes	[6, 7, 33, 78]
Race	Yes	[78]

Some studies have proposed the male gender as a predictor for sepsis post-trauma.[6,7,33,78] After trauma, the continuous increase in cytokines and the subsequent immunosuppression make the body prone to sepsis. A study performed on animals showed that pro-estrus females are not immunode-pressed compared with male and ovariectomized mice after trauma.[79] Other test results have demonstrated that estrogen produces beneficial effects on immune and cardiovascular function after trauma[80] by reducing the release of cytokine production, such as TNF-α, and maintaining the immune response.[81] Thus, estrogen plays an important role in the gender dimorphism of post-traumatic sepsis.

African American race is reported as a risk factor of sepsis following trauma.[78] However, there has been no extensive research conducted to investigate the role of racial or ethnic factors in post-traumatic sepsis. More research is warranted to explore the association between ethnicity and this complication.

### Injury characteristics

Injury severity, mechanism of injury, number of injuries, hypotension on admission, and other injury characteristics are factors associated with post-traumatic sepsis.

Trauma can cause deficits in the immune system by depressing the humoral and cell-mediated systems. After major trauma, the function of lymphocytes is depressed. The neutrophil chemotaxis is decreased and monocyte antigen-presenting capacity is impaired. There are also changes in complement components.[82] Different degrees of trauma severity may lead to the different influences on immune function. The main measures of injury severity are trauma-scoring systems. Trauma scoring systems are divided into 3 categories: Anatomical scoring systems, physiological scoring systems and combined scoring systems.[83]

The calculation of most trauma scoring systems is time consuming and complicated. However, among the various scoring systems, the Injury Severity Score (ISS) and the New Injury Severity score (NISS) can be rapidly calculated and are most widely used in predicting outcomes of trauma patients. The ISS and NISS are members of anatomical scoring systems. The ISS is based on the Abbreviated Injury Scale (AIS) severity values, and it was first developed in 1974.[84] It is calculated as the sum of the squares of the highest AIS values from each of the three most severely impaired body regions. It has some limitations, for example, it does not represent multiple injuries in the same body region and it considers injuries with an equal AIS score to be the same severity regardless of the injured body region.[85] The NISS was proposed by Osler *et al.* in 1997 to counter the limitations of the ISS.[86] It is calculated as the sum of squares of the three most severe injuries, regardless of the body region injured. The ISS or NISS ranges from 1 to 75. Increasing injury severity measured by the ISS and NISS was associated with increased incidence of sepsis.[3,6,7,78,87]

In addition to the ISS and NISS, the Glasgow coma scale (GCS), which assesses the level of clinical consciousness is also a predictor of sepsis.[6,7] The GCS was first described by Teasdale and Jennett in 1974.[88] It is the sum of 3 components that describes a patient’s best motor response, verbal response and eye opening to stimuli. It ranges from 3 to 15, and the lower score the patient receives, the worse condition the patient is in. The GCS belongs to physiological scoring systems.[89]

The anatomy scoring systems such as the ISS and NISS represent the physical degeneration of the body, and the physiological scoring systems, such as the GCS, stand for the physiological impairment caused by trauma. Compared to biomarkers, obtaining the indices of these scoring systems is easier, earlier and cheaper. Further work may be needed to verify the accuracy of the scoring systems and to explore whether the combination of the two types of scoring systems can improve the predictive power for sepsis in trauma patients.

There are several injury characteristics reported as risk factors, such as number of red blood cell units transfused,[3] hypotension on emergency department presentation[78] and number of injuries.[6] However, studies on these factors are not common.

## Conclusion

Early prediction of sepsis development and early intervention for patients at risk can decrease the morbidity and mortality after trauma. The prediction of sepsis in trauma patients is still a challenge. Though approximately 180 biomarkers for sepsis have been reported, the studies performed on the biomarkers for post-traumatic sepsis are few and the results are controversial. Trauma can affect immunologic function, and injury characteristics, such as injury severity and the number of injuries, are risk factors that are associated with sepsis following trauma. To trauma patients, demographic variables, including age, gender and race are also risk factors. Obtaining the information of injury characteristics and patient demographics is earlier, easier and cheaper than biomarkers, but the connection between these factors and the pathophysiology of sepsis is yet to be identified or clarified. Additional work is needed to verify the predictors and find more efficient and accurate ways that can better predict sepsis in trauma patients.

## Call for Papers!

Burns & Trauma, an Open Access journal focusing on latest and best achievements in basic, clinical and translational research related to burns and trauma, warmly invite you to submit manuscripts on the upcoming **special issue—Biomaterial Research**, which will be published in **October, 2014**. We guarantee timely response to submissions within two weeks.

Submit your paper: http://www.journalonweb.com/bt/
